# Cerebellar Excitability Regulates Physical Fatigue Perception

**DOI:** 10.1523/JNEUROSCI.1406-22.2023

**Published:** 2023-04-26

**Authors:** Agostina Casamento-Moran, Ronan A. Mooney, Vikram S. Chib, Pablo A. Celnik

**Affiliations:** ^1^Department of Physical Medicine and Rehabilitation, Johns Hopkins University, Baltimore, Maryland 21287; ^2^Kennedy Krieger Institute, Baltimore, Maryland 21287; ^3^Department of Biomedical Engineering, Johns Hopkins University, Baltimore, Maryland 21287; ^4^Department of Neuroscience, Johns Hopkins University, Baltimore, Maryland 21287

**Keywords:** cerebellum, fatigability, fatigue, interoception, motor control, TMS

## Abstract

Fatigue is the subjective sensation of weariness, increased sense of effort, or exhaustion and is pervasive in neurologic illnesses. Despite its prevalence, we have a limited understanding of the neurophysiological mechanisms underlying fatigue. The cerebellum, known for its role in motor control and learning, is also involved in perceptual processes. However, the role of the cerebellum in fatigue remains largely unexplored. We performed two experiments to examine whether cerebellar excitability is affected after a fatiguing task and its association with fatigue. Using a crossover design, we assessed cerebellar inhibition (CBI) and perception of fatigue in humans before and after “fatigue” and “control” tasks. Thirty-three participants (16 males, 17 females) performed five isometric pinch trials with their thumb and index finger at 80% maximum voluntary capacity (MVC) until failure (force <40% MVC; fatigue) or at 5% MVC for 30 s (control). We found that reduced CBI after the fatigue task correlated with a milder perception of fatigue. In a follow-up experiment, we investigated the behavioral consequences of reduced CBI after fatigue. We measured CBI, perception of fatigue, and performance during a ballistic goal-directed task before and after the same fatigue and control tasks. We replicated the observation that reduced CBI after the fatigue task correlated with a milder perception of fatigue and found that greater endpoint variability after the fatigue task correlated with reduced CBI. The proportional relation between cerebellar excitability and fatigue indicates a role of the cerebellum in the perception of fatigue, which might come at the expense of motor control.

**SIGNIFICANCE STATEMENT** Fatigue is one of the most common and debilitating symptoms in neurologic, neuropsychiatric, and chronic illnesses. Despite its epidemiological importance, there is a limited understanding of the neurophysiological mechanisms underlying fatigue. In a series of experiments, we demonstrate that decreased cerebellar excitability relates to lesser physical fatigue perception and worse motor control. These results showcase the role of the cerebellum in fatigue regulation and suggest that fatigue- and performance-related processes might compete for cerebellar resources.

## Introduction

Fatigue, the subjective sensation of weariness, increased sense of effort, or exhaustion, is one of the most common and debilitating symptoms across neurologic and chronic illnesses (Kluger et al., 2013; Penner and Paul, 2017). In addition, fatigue has major clinical significance across neuropsychiatric diseases, such as major depressive disorder, as it represents an important risk factor for depression (Skapinakis et al., 2004; Stephan et al., 2016). Despite its epidemiological importance, there are limited effective treatments or rehabilitation strategies for fatigue (Kluger et al., 2013). This is because of our limited understanding of fatigue, which results from inappropriately interchanging the concept of fatigue (i.e., the perception) with fatigability (i.e., the objective decrements in motor or cognitive performance over time) (Kluger et al., 2013). While we have an extensive understanding of the fatigability-related changes that occur at the muscular (Hunter et al., 2004; Enoka and Duchateau, 2008), spinal (Gandevia, 2001; Klass et al., 2008), and supraspinal (Gandevia, 2001) levels, little is known about the neurophysiological mechanisms of fatigue (i.e., the percept). Recent theoretical accounts, however, propose that fatigue arises as an affective response from the inability to maintain bodily homeostasis (Stephan et al., 2016), and that deactivation in cortical and subcortical areas calibrates the sensorimotor state to help maintain homeostasis under fatiguing conditions (Hogan et al., 2020).

Growing evidence suggests that the cerebellum, primarily known for its role in optimizing motor control and learning (Bastian, 2006; Manto et al., 2012), is a key structure for nonmotor functions (Strick et al., 2009; Gill and Sillitoe, 2019; Schmahmann et al., 2019) and perceptual processes, such as nociception, self-motion, timing, vision, and audition (Baumann et al., 2015). However, the role of the cerebellum in fatigue perception remains largely unexplored. The “universal cerebellar transform theory” proposes that, by integrating internal representations with external stimuli, the cerebellum maintains behavior around a homeostatic baseline (Schmahmann et al., 2019). It is possible, therefore, that the cerebellum aids this process by updating the priors regarding the sensorimotor state (Schutter, 2016), which in turn could minimize fatigue perception.

In this study, we examined whether the cerebellum plays a modulatory role under fatiguing conditions and, if so, whether it is capable of simultaneously regulating fatigue perception and motor performance or whether these two processes compete for cerebellar resources. Specifically, we examined how cerebellar excitability is modulated after a fatiguing task and its impact on fatigue perception and motor control. While there is no direct empirical evidence linking the cerebellum with fatigue perception, the cerebellum is thought to play a key regulatory role when fatiguing conditions occur through error-based learning (Barash et al., 1999). Furthermore, evidence suggests that the cerebellum plays a modulatory role in nociception (i.e., the perception of pain), in that lower cerebellar activation relates to milder pain perception (Moulton et al., 2010). Consequently, we hypothesized that, if indeed the cerebellum plays a modulatory role under fatiguing conditions, cerebellar excitability will be reduced after a fatiguing task and associated with milder fatigue perception. We also reasoned that reduced cerebellar excitability, while minimizing the perception of fatigue, would be associated with poor motor control because ample evidence suggests that motor control (Gates and Dingwell, 2008; Missenard et al., 2008; Lohse and Sherwood, 2012; Monjo et al., 2015; Hunter, 2018; Bächinger et al., 2019) and learning (Branscheidt et al., 2019) are impaired after fatiguing tasks. We tested these two hypotheses in two separate experiments assessing the relationship between cerebellar excitability, fatigue, and motor control before and after performing a fatiguing task.

## Materials and Methods

### Participants

A total of 33 healthy young adults (mean age 27 years; age range 18-35 years; 16 males; 28 right-handed) participated in the study. We screened participants before enrollment for contraindications to transcranial magnetic stimulation (TMS) using a questionnaire, none of them suffered from any neurologic or psychiatric disorder, nor were they taking any centrally acting prescribed medication. The study was approved by the Johns Hopkins School of Medicine Institutional Review Board in accordance with the declaration of Helsinki and we obtained written informed consent from each participant.

### Overview of the experimental paradigms

#### Experiment 1

##### Modulation of cerebellar, corticomotor, and intracortical excitability after a fatiguing task

**Figure 1.**
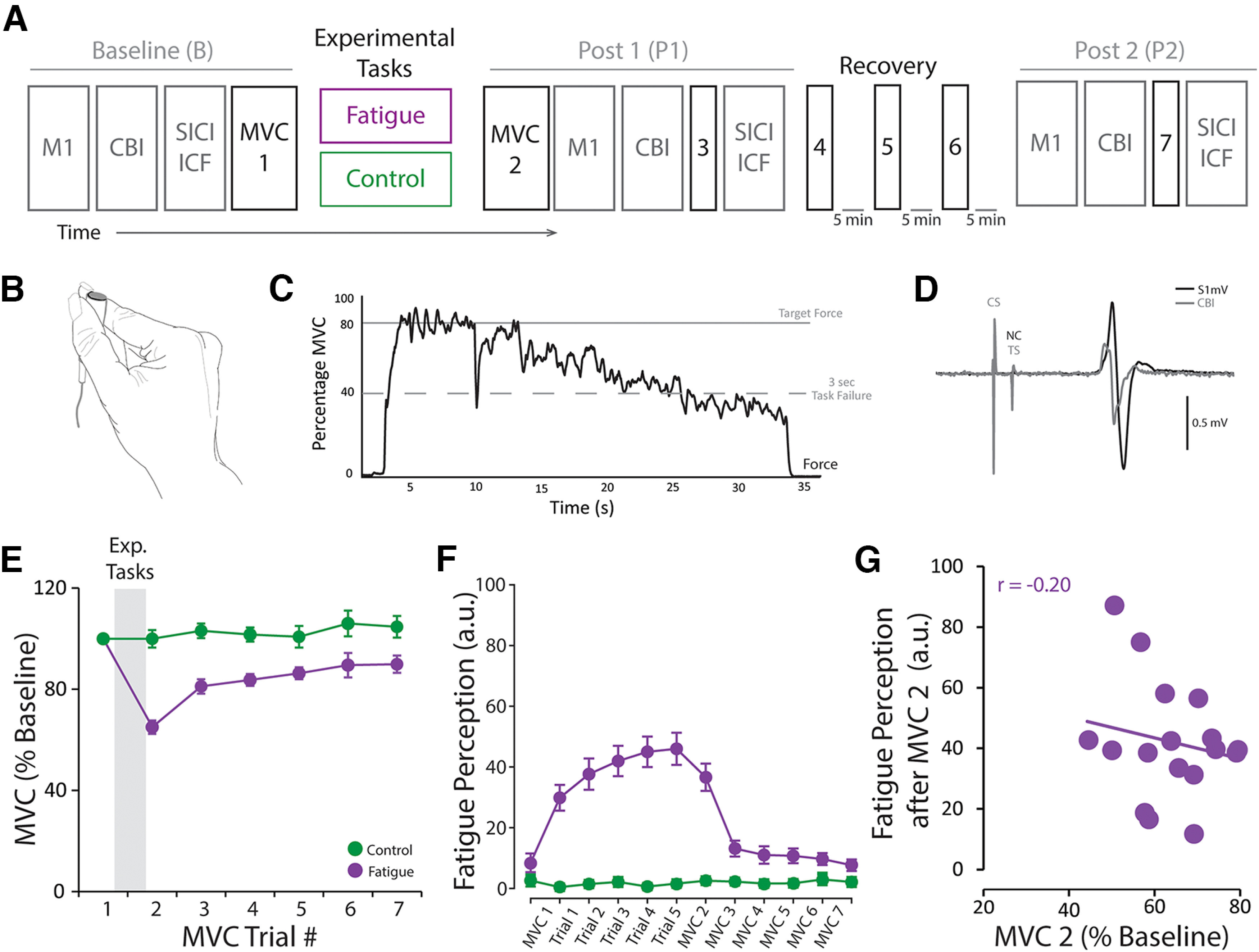
Design of Experiment 1. ***A***, Experiment 1 consisted of two nonconsecutive sessions separated by at least 24 h. Each session was either a fatigue or a control one, and the order was pseudo-randomized across participants. At the beginning of all sessions, we obtained baseline (B) measures of corticomotor excitability (M1), cerebellar brain inhibition (CBI), intracortical inhibition (SICI, ICF), and strength (MVC). Participants then performed one of the experimental tasks (i.e., fatigue or control) where we obtained measures of both fatigue and fatigability. The fatigue task consisted of 5 TTF trials, whereas the control task consisted of 5 constant isometric low-force trials. Right after the experimental tasks, we assessed MVC, M1, CBI, SCI, and ICF to examine the immediate effects of fatigue and fatigability on supraspinal excitability (P1). To understand the time course of these changes, participants sat comfortably and rested for 15 min (Recovery). We monitored participants recovery by obtaining MVC and fatigue ratings every 5 min. Last, we repeated all neurophysiological measures (P2) to examine the “longer-term” effects of fatigue and fatigability on supraspinal excitability. ***B***, Graphical representation of the pinch task that participants used to perform the fatigue and control experimental tasks. ***C***, Graphical representation of a TTF trial. Participants had to match the target force (80% MVC) for as long as possible while the experimenter was verbally encouraging them to do so. The trial was terminated when the force exerted decayed <40% MVC for 3 consecutive seconds (i.e., task failure point). ***D***, Example electromyography traces represent the MEPs used to quantify CBI. First, we determined the stimulus intensity required to elicit an ∼1 mV MEP (S1mV, NC). To assess CBI, a subthreshold CS set to 5% MSO below brainstem threshold was delivered over ipsilateral cerebellar cortex 5 ms before a suprathreshold TS set to S1mV delivered over contralateral M1. We quantified CBI as the ratio MEP amplitude between the single and paired-pulse MEPs. ***E***, The fatigue task (purple) significantly reduced participant's MVC throughout the length of the experimental session, whereas MVC remained unchanged after the control task (green). ***F***, The fatigue task (purple) significantly increased participant's fatigue perception throughout the length of the experimental session, whereas fatigue perception remained low and unchanged after the control task (green). ***G***, Fatigue (perception) and fatigability (i.e., % MVC after the fatigue task) are independent. In time-course result plots, circles represents the average for each session at each time point, and error bars represents the SEM across participants.

The overall experimental design can be found in Figure 1*A*. Eighteen healthy young adults (mean age 26.3 years; age range 18-35 years; 10 males; 15 right-handed) participated in Experiment 1. There were two experimental sessions, separated by a minimum of 24 h. In each session, participants were pseudo randomly allocated to complete the fatigue or control task with their dominant hand, in a repeated-measures crossover design. Measures of cerebellar, corticomotor, and intracortical excitability were assessed at baseline (B) and at two time points after (P1 and P2) the tasks using TMS. We collected motor-evoked potential (MEP) amplitude data (i.e., corticomotor excitability) from all 18 participants. Because CBI is not always tolerated by participants, we collected CBI data from 12 of the 18 participants. Last, we collected SICI and ICF data (i.e., intracortical excitability) from 15 of the 18 participants recruited in Experiment 1.

#### Experiment 2

##### Behavioral consequences of reduced cerebellar excitability after a fatiguing task

The overall experimental design can be found in Figure 3*A*. We examined the behavioral consequences of reduced cerebellar excitability after a fatiguing task by measuring cerebellar and corticomotor excitability as well as performance during a motor control and an active proprioceptive task (i.e., tasks that rely heavily on cerebellar function) before and after the fatigue and control tasks. Twenty healthy young adults (mean age 27.5 years; age range 19-35 years; 9 males; 16 right-handed) participated in Experiment 2. Five of the participants in this experiment also participated in Experiment 1. As in Experiment 1, there were two experimental sessions separated by a minimum of 24 h. In each session, participants were pseudo randomly allocated to complete a fatigue or control task with their dominant hand (see above for details about the tasks), in a repeated-measures crossover design. We collected behavioral data from all 20 participants. Because CBI is not always tolerated by participants, we collected CBI and MEP amplitudes from 12 of the 20 participants. To assess motor control, participants performed a total of 7 blocks of 10 fast-reverse at target goal-directed trials. Specifically, they performed three familiarization blocks to ensure that participants understood and could perform the task; 2 baseline blocks (Pre) used as a measure of performance before the fatigue and control tasks; and 2 blocks after the tasks (Post) to examine the effect of the fatigue task on motor control. Last, to assess proprioception, participants performed a total of 9 trials (three trials per target position) before and after the fatigue and control tasks.

### Experimental procedures

#### Maximal voluntary capacity (MVC)

Participants held the force transducer between their thumb and index fingers of their dominant hand (see Fig. 1*B*), increased their force to their maximum, and maintained it for 3 s. The index finger was in a semi-flexed flexed position that facilitated the griping of the force sensor between the two digits. They exerted MVC contractions before (pre-MVC) and after (post-MVC) the fatigue and control tasks (for more details on these two experimental tasks, see below). We used the pre-MVC to quantify the target forces for each of the tasks and to obtain the participants' initial voluntary force capacity. Participants repeated three MVC trials to ensure the accurate measurement of the participants' initial MVC. We used the post-MVCs to determine the degree of fatigability induced by the experimental tasks as well as to monitor the recovery along the experimental sessions. All post-MVCs were single trials, because, by then, participants were familiarized with the task/force sensor and we wanted to minimize the fatigue induced by having to exert MVC trials.

#### Experimental tasks: fatigue and control

Participants held the force transducer between their thumb and index fingers of their dominant hand (see Fig. 1*B*) and sat in front of a computer monitor that displayed their target force. As during the MVC task, the index finger was in a semi-flexed flexed position that facilitated the griping of the force sensor between the two digits. Force was exerted by pressing the force sensor against the thumb with index finger abduction. The target forces were 80% and 5% of participants' pre-MVC for the fatigue and control tasks, respectively. During the fatigue task, participants performed five isometric time-to-task failure (TTF) trials that required them to match the target force for as long as possible while the experimenter was verbally encouraging them to do so. The trial was terminated when the force exerted decayed <40% MVC for 3 consecutive seconds (Hunter, 2018) (i.e., task failure point; see Fig. 1*C*). Importantly, we did not inform participants regarding the criteria that terminated a trial, and the participants did not report having figured it out. On the other hand, during the control task, participants performed five constant isometric low-force trials that require them to match the target force for 30 s.

#### Fatigue rating scale

After each exertion trial (i.e., MVC, TTF, or isometric low-force trials), we assessed participants' perception of fatigue through a computerized fatigue rating scale that asked, “*How much Fatigue do you feel?”* To answer, participants use the right-left arrows of the keyboard to displace a cursor horizontally through a line that converted their responses into numerical ones (ranging from 0 to 100). Throughout the number line, we provided six levels of fatigue as the mean to give participants a reference to the meaning of those numbers: *“No Fatigue”* (i.e., 0), *“Mild Fatigue”* (i.e., 20), *“Moderate Fatigue”* (i.e., 40), *“Severe Fatigue”* (i.e., 60), *“Very Severe Fatigue”* (i.e., 80), and *“Worst Fatigue Possible”* (i.e., 100).

#### Cerebellar, corticomotor, and intracortical excitability

##### Measuring surface electromyography (EMG)

Surface EMG was recorded from the first dorsal interosseous (FDI) of the dominant hand using 25 mm^2^ Ag-AgCl recording electrodes (Vermed), with a ground electrode positioned on the distal head of the ulna. Specifically, and as previously done in the laboratory (Branscheidt et al., 2019; Mooney et al., 2021), the bipolar electrodes were placed in the belly of the FDI and in the first distal phalange if the index finger. EMG signals were amplified (1000×) and bandpass filtered (10-1000 Hz) using an AMT-8 amplifier (Bortec Biomedical), sampled at 5 kHz using a Micro1401-4 interface (CED) and recorded with Signal software (Version 4.02; CED).

##### TMS of M1

TMS was applied using a 70-mm-diameter figure-of-8 coil connected to a single monophasic Magstim 200^2^ magnetic stimulator or two monophasic Magstim 200^2^ magnetic stimulators via a Bistim^2^ module (Magstim). The coil was held tangentially to the scalp (∼45° to the mid-sagittal line) to induce posterior-anterior current in the brain. The optimal site to elicit a consistent MEP in the FDI muscle was identified and marked over M1 and kept constant throughout using Brainsight neuronavigation (Rogue Research).

##### TMS of the brainstem and cerebellum

TMS was applied using a 110-mm-diameter cone coil connected to a monophasic Magstim 200^2^ magnetic stimulator. For brainstem stimulation assessing pyramidal tract activation, the coil was held over the inion. For cerebellar stimulation, the coil was held 3 cm lateral to the inion over the cerebellar cortex ipsilateral to the dominant hand. The current in the coil was directed downward to induce upward current in the cerebellar cortex.

##### Assessing cerebellar and corticomotor excitability

First, we determined brainstem threshold, which was defined as the nearest 5% maximum stimulator output (MSO) that elicited an MEP of at least 50 µV in amplitude in 5 of 10 trials with FDI preactivated through a low-level voluntary contraction (∼10% of MVC) (Ugawa et al., 1995). Since no MEPs were evoked via brainstem pathways at 75% maximum stimulator output, we used this intensity as the brainstem threshold for all participants (Spampinato and Celnik, 2017). Next, we determined the stimulus intensity required to elicit an ∼1 mV MEP with FDI at rest (S1mV, nonconditioned). To measure cerebellar inhibition (CBI), we delivered a subthreshold conditioning stimulus (CS) set to 5% MSO below brainstem threshold over ipsilateral cerebellar cortex 5 ms before a suprathreshold test stimulus (TS) set to S1mV delivered over contralateral M1 (see Fig. 1*D*). At baseline, we delivered 12 trials for each condition (nonconditioned, CBI; 24 trials total) in a randomized order. At each post time point, we collected 12 trials at the baseline S1mV intensity to assess changes in corticomotor excitability. If necessary, we adjusted the intensity of the TS to maintain an ∼1 mV nonconditioned MEP before collecting the CBI trials at the different post time points. The S1mV and nonconditioned MEPs for each session and time point can be found in Table 1. It is important to highlight that, despite the relatively low tolerability of CBI, CBI as a technique has low measurement error (∼15%) within and between sessions, and its smallest detectable change (SDC) does not change significantly with sample sizes >10 participants (Mooney et al., 2021).

**Table 1.** Neurophysiological results and stimulation parameters from Experiments 1 and 2*^a^*

	Fatigue			Control		
	B	P1	P2	B	P1	P2
Experiment 1						
MEP (mV)	1.24 (0.11)	0.44 (0.06)	0.65 (0.08)	1.17 (0.07)	0.78 (0.09)	0.83 (0.11)
CBI S1mV (% MSO)	63 (2)	72 (4)	70 (4)	63 (2)	70 (3)	64 (2)
CBI NC MEP (mV)	1.24 (0.11)	1.26 (0.10)	1.28 (0.15)	1.17 (0.07)	1.22 (0.11)	1.11 (0.09)
AMT (% MSO)	49 (2)	—	—	49 (2)	—	—
SICI/ICF S1mV (% MSO)	72 (2)	80 (3)	79 (3)	71 (2)	73 (2)	73 (2)
SICI/ICF NC MEP (mV)	1.07 (0.09)	1.12 (0.12)	1.05 (0.10)	1.09 (0.08)	1.11 (0.11)	1.09 (0.11)
SICI (C/NC)	0.29 (0.05)	0.47 (0.08)	0.39 (0.06)	0.3 (0.06)	0.38 (0.07)	0.36 (0.05)
ICF (C/NC)	1.13 (0.11)	1.22 (0.09)	1.13 (0.07)	1.13 (0.07)	1.04 (0.09)	1.18 (0.11)
Experiment 2						
MEP (mV)	1.03 (0.12)	0.73 (0.14)	—	1.19 (0.14)	0.75 (0.22)	—
CBI S1mV (% MSO)	58 (3)	60 (3)	—	58 (4)	60 (4)	—
CBI NC MEP (mV)	1.03 (0.12)	1.01 (0.10)	—	1.19 (0.14)	1.17 (0.14)	—

*^a^*Data are group mean (SEM). NC, Nonconditioned.

##### Assessing intracortical excitability

We first determined the FDI active motor threshold (AMT), which was defined as the stimulus intensity required to elicit an MEP of at least 200 µV in amplitude in 5 of 10 trials with FDI preactivated through a low-level voluntary contraction (∼10% of MVC) (Rossini et al., 2015). Next, we redetermined S1mV to account for lower power output with the coil connected to the Bistim^2^ module. To assess short interval intracortical inhibition (SICI) and intracortical facilitation (ICF), we delivered a subthreshold CS set to 80% AMT 3 and 12 ms before a suprathreshold TS set to S1mV (Kujirai et al., 1993). At baseline, we delivered 12 trials for each condition (nonconditioned, SICI, ICF; 36 trials total) in a randomized order. If necessary, we adjusted the intensity of the TS to maintain an ∼1 mV nonconditioned MEP before collecting the 36 trials at P1 and P2 (Table 1).

#### Motor control and proprioception

To examine the motor control consequences of reduced cerebellar excitability after a fatiguing task, we quantified endpoint error during fast reverse-at-target goal-directed movements with the dominant index finger. We specifically chose this task because it requires the preplanned coordination of the antagonistic muscles, which relies heavily on cerebellar function (Lang and Bastian, 1999; Köster et al., 2002; Casamento-Moran et al., 2017, 2020) and minimizes the influence of fatigue-induced tremor on endpoint error (Casamento-Moran et al., 2020). Fast reverse-at-target goal-directed movements involved accurately matching the peak displacement and the time-to-peak displacement to a target with index finger adduction and abduction movements (see Fig. 5*A*). The movements were unloaded, the target peak displacement was 8°, and the target time-to-peak displacement was 160 ms. During the task, the participant sat comfortably in an upright position and faced a monitor located at eye level. The participant's dominant elbow was flexed to ∼90°, the wrist was in a pronated position, and the hand rested flat over a customized 3D-printed device. The hand was covered by an opaque structure to prevent participants from using visual feedback during the task.

To examine the proprioceptive consequences of reduced cerebellar excitability after a fatiguing task, we quantified matching error during a position matching task with the dominant and nondominant index fingers. We specifically chose this task because proprioceptive acuity during active position matching tasks depends on the integrity of the cerebellum (Bhanpuri et al., 2012, 2013). The position matching task involved the following: (1) displacing the reference digit (i.e., the nondominant index finger) to match the target position displayed as a horizontal line on the monitor; and (2) displacing the indicator digit (i.e., dominant index finger) to match the perceived position of the reference digit without any visual cues. The trial was terminated once the participant perceived that both digits had similar positions. The movements were unloaded, and there were three target positions (5, 10, or 15 degrees). During the task, the participant sat comfortably in an upright position and faced a monitor located at eye level. Participants' dominant and nondominant elbows were flexed to ∼90°, the wrists were in a pronated position, and the hands rested flat over two customized 3D-printed devices. The hands were covered by two opaque structures to prevent participants from using visual feedback during the task.

For both tasks, the axis of rotation of the customized hand devices was in line with the axis of rotation of the metacarpophalangeal joint of the index fingers. We measured the displacement of the index finger using a low-friction potentiometer (SP22G-5 K, Mouser Electronics), located directly inferior to the metacarpophalangeal joints. The sampling frequency of displacement signals was 1000 Hz (NI-DAQ card, model USB-6009, National Instruments), and these signals were stored on a personal computer.

### Data handling and analysis for Experiments 1 and 2

#### Fatigue

We used the fatigue rating obtained after MVC2 (i.e., right after the experimental tasks) as our metric of fatigue perception after the experimental tasks (i.e., fatigue/control). In addition, we examined the change in fatigue perception with Equation 1 as follows:

(1) $$ChangeinFatigue=FatigueRating_{MVC2}-FatigueRating_{MVC1}$$

#### Fatigability

We measured fatigability by examining declines in force capacity (i.e., MVC) and TTF. We examined participants' force capacity throughout the experimental sessions by looking at their raw and normalized MVC. We quantified changes in participants' force capacity by normalizing their MVC to the baseline MVC trial performed during each experimental session (MVC1; Eq. 2).

(2) $$MVC\left(\text{\% Baseline}\right)=\frac{MVC*100}{MVC1}$$

TTF represents the time it took for the force exerted by the participant to decay <40% MVC for 3 consecutive seconds. The average TTF (across the 5 TTF trials) for Experiment 1 was 42.9 ± 17.0 s, while for Experiment 2 it was 44.3 ± 10.0 s.

### Neurophysiological data

Trials contaminated by prestimulus EMG activity (root mean squared EMG >10 μV; 100 ms before stimulation) were discarded. For all muscles, trials with MEP amplitudes >2 SDs outside the mean were also discarded (Experiment 1: 4.2% and 3.7% of the total trials were discarded in the fatigue and control sessions, respectively; Experiment 2: 0.7% and 0.65% of the total trials were discarded in the fatigue and control sessions, respectively). All participants had at least 10 valid trials per condition. To quantify CBI, SICI, and ICF, conditioned MEP amplitudes were expressed as a ratio of the nonconditioned MEP amplitude at each time point (Table 1). Last, we examined the change in cerebellar excitability with Equations 3 and 4 for Experiments 1 and 2, respectively:

(3) $$Exp1\Delta CBI=CBI_{P1}-CBI_{B}$$

(4) $$Exp2\Delta CBI=CBI_{Post}-CBI_{Pre}$$

#### Motor control

We quantified endpoint error as the hypotenuse of the normalized position and temporal errors (Eq. 5), which represents the shortest distance from the target. Specifically, position error was the absolute vertical deviation from the targeted peak displacement, and temporal error was the absolute horizontal deviation from the targeted peak displacement. We normalized both errors to the appropriate targets (Eq. 6, 7) as follows:

(5) $$Endpointerror(\%)=\sqrt{{\left(positionerror\right)}^{2}+{\left(timeerror\right)}^{2}}$$

(6) $$Psitionerror(\%)=\frac{abs(peakdisplacementerror\left(degrees\right))}{targetedpeakdisplacement(degrees)}\times 100$$

(7) $$Temporalerror(\text{\%})=\frac{abs(timetopeakdisplacementerror\left(ms\right))}{targ\text{e}tedtimetopeakdisplacement(ms)}\times 100$$

We quantified endpoint variability with the SD of the endpoint error from trial to trial.

#### Proprioception

We quantified matching error as the absolute difference between the reference and indicator positions. The position of each digit was quantified as the average displacement (in degrees) ±1 s around the point at which participants terminated the trial (Eq. 8).

(8) Matchingerror(°)=|ReferencePosition(°)−IndicatorPosition(°)|

### Statistical analysis

All statistical analyses were performed using SPSS software (version 25, IBM). Normality was assessed using Shapiro–Wilk's test. Non-normal data (MEP amplitude in Experiment 2) were log-transformed for statistical analysis. The significance level was set at *p* < 0.05. Group data are presented as mean ± SEM.

#### Experiment 1

We analyzed the MVC data using two-way repeated-measures ANOVAs with TASK (fatigue, control) and TIME (MVC1 to MVC7; 7 time points) as the within-subjects' factors as well as the neurophysiological data (CBI, MEP amplitude, SICI, and ICF) using separate two-way repeated-measures ANOVAs with TASK (fatigue, control) and TIME (B, P1, P2) as the within-subjects' factors. We used Greenhouse–Geiser correction to adjust for lack of sphericity in the repeated-measures ANOVA examining changes in MEP amplitude. *Post hoc* comparisons were made using two-tailed paired *t* tests and were adjusted when necessary, using the Bonferroni correction. We used paired *t* test to compare the perception of fatigue after each of the tasks (i.e., Fatigue_MVC2_) and Pearson correlation analyses to determine associations between fatigue (Fatigue_MVC2_) perception, change in fatigue perception (Fatigue_MVC2_ – Fatigue_MVC1_), TTF, MVC, CBI, change in CBI (CBI_P1_ – CBI_B_), and MEP amplitude.

#### Experiment 2

We analyzed the MVC data using two-way repeated-measures ANOVAs with TASK (fatigue, control) and TIME (MVC1 to MVC3; 3 time points) as the within-subjects' factors as well as the neurophysiological data (CBI and MEP amplitude) using separate two-way repeated-measures ANOVAs with TASK (fatigue, control) and TIME (Pre and Post) as the within-subjects' factors. Behavioral data (Endpoint and Position-Matching error) were analyzed with separate two-way repeated-measures ANOVAs with TASK (fatigue, control) and TIME (Pre and Post) as the within-subjects' factors. *Post hoc* comparisons were made using two-tailed paired *t* tests and were adjusted when necessary, using the Bonferroni correction. We used paired *t* test to compare the perception of fatigue after each of the tasks (i.e., Fatigue_MVC2_) and Pearson correlation analyses to determine associations between fatigue (Fatigue_MVC2_) perception, change in fatigue perception (Fatigue_MVC2_ – Fatigue_MVC1_), TTF, MVC, CBI, change in CBI (CBI_Post_ – CBI_Pre_), and MEP amplitude.

## Results

### Experiment 1: Modulation of cerebellar, corticomotor, and intracortical excitability after a fatiguing task

#### Fatigue and fatigability

We examined whether our fatigue task reliably induced fatigue and fatigability. We used participants' MVC (normalized to baseline) to quantify the level of fatigability (Fig. 1*E*). We found a significant main effect of TASK (*F*_(1,17)_ = 22.05, *p* < 0.001) demonstrating that participants exhibited lower force capacity during the fatigue session compared with the control one. Importantly, we also found a significant TASK × TIME interaction (*F*_(6,102)_ = 11.08, *p* < 0.001). *Post hoc* analysis demonstrated that, while baseline force capacity was similar between sessions, the fatigue task significantly reduced participant's force capacity and the control task did not. We obtained the same results when using Raw MVC values. Specifically, we found a significant main effect of TASK (*F*_(1,17)_ = 11.36, *p* = 0.004) and a significant TASK × TIME interaction (*F*_(6,102)_ = 11.73, *p* < 0.001) demonstrating similar patterns. Together, these results suggest that, regardless of how we quantify a participant's force capacity (i.e., raw or normalized MVC), the fatigue task induced fatigability while the control task did not.

On the other hand, at baseline, perception of fatigue was similar between the fatigue (9.64 + 3.2) and control (3.78 + 2.03) sessions (*t*_(1,17)_ = −1.42, *p* = 0.18; Fig. 1*F*). In addition, fatigue perception was significantly lower than mild in both experimental sessions (fatigue: *t*_(1,17)_ = −3.23, *p* = 0.007; control: *t*_(1,17)_ = −7.98, *p* < 0.001). However, we found that participants fatigue ratings were significantly higher after the fatigue task compared with the control one (fatigue: 37.8 + 4.67, control: 3.73 + 1.5, *t*_(1,17)_ = 7.93, *p* < 0.001, Fig. 1*F*). These results confirm that our fatigue task induced both fatigability and fatigue.

Fatigue should not be confused with fatigability (Kluger et al., 2013). While fatigue refers to the subjective sensation of weariness, increased sense of effort, or exhaustion, fatigability refers to objective decrements in performance over time (Kluger et al., 2013). Fatigue and fatigability are not only distinct but potentially independent phenomena (Kluger et al., 2013). To understand the relation between fatigue and fatigability in our study, we examined the correlation between fatigue ratings, reduced MVC, and average TTF. We found that neither the reduced MVC (*r* = −0.20, *p* = 0.42; Fig. 1*G*) nor the TTF (*r* = 0.34, *p* = 0.17) related with fatigue ratings, supporting the idea that fatigue and fatigability are independent phenomena with different underlying mechanisms.

#### Reduced cerebellar excitability relates to milder fatigue

**Figure 2.**
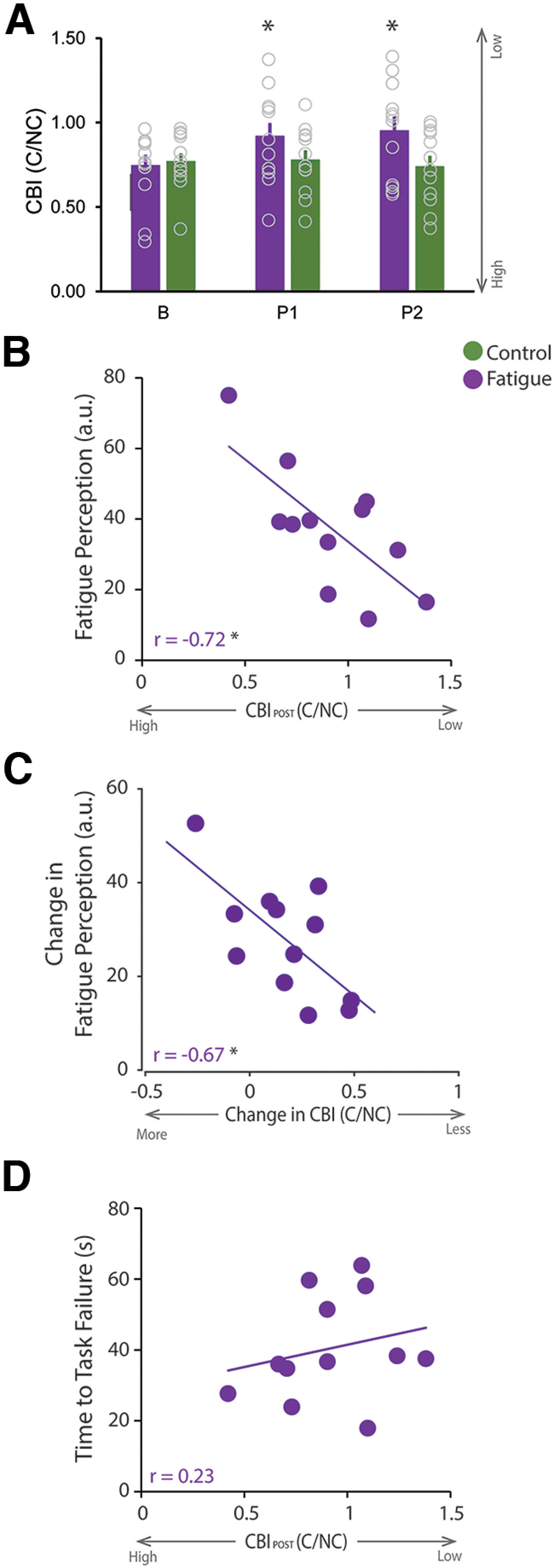
Reduced CBI relates to a milder perception of fatigue in Experiment 1. ***A–D***, Purple represents data during the fatigue session. Green represents data during the control session. For all bar graphs, the bar represents the average for each condition at each time point, with individual data represented by the open circles, and the error bar represents the SEM across participants. ***A***, CBI was significantly reduced immediately (P1) and 30 min after (P2) the fatigue task. In contrast, CBI was not altered by the control task. ***B***, CBI_P1_ was significantly and negatively correlated with fatigue ratings. ***C***, Change in CBI (P1 relative to ***B***) was significantly and negatively correlated with the change in fatigue ratings. ***D***, CBI_P1_ did not correlate with TTF (i.e., fatigability). *p < 0.05.

The main goal of the first experiment was to examine changes in cerebellar excitability and its relation to fatigue. We measured CBI before and after the experimental tasks to examine whether our fatiguing task modulated cerebellar excitability. We found a significant TASK × TIME interaction (*F*_(2,22)_ = 3.48, *p* = 0.048; Fig. 2*A*), and *post hoc* analysis demonstrated that CBI was significantly reduced immediately (P1) and 30 min after (P2) the fatigue task. In contrast, CBI was not altered by the control task. Of note, the fatigue-related change in CBI (ΔCBI_Fat_: 0.17) is greater than the known measurement noise of the technique (SDC = 0.081) (Mooney et al., 2021).

Since our fatigue task significantly induced both fatigability and fatigue, as demonstrated with reduced MVC (Fig. 1*E*) and greater fatigue ratings (Fig. 1*F*) compared with the control task, we then examined whether reduced cerebellar excitability relates to fatigue and fatigability by examining the correlation between CBI_P1,_ fatigue ratings, reduced MVC, and average TTF after the fatigue task. We found that CBI_P1_ was significantly and negatively correlated with fatigue ratings (*r* = −0.72, *p* = 0.008; Fig. 2*B*), but not with reduced MVC (*r* = 0.20, *p* = 0.53) nor TTF (*r* = 0.23, *p* = 0.47, Fig. 2*D*). Furthermore, we found that a greater change in CBI was significantly and negatively correlated with a smaller change in fatigue (perception) ratings (*r* = −0.67, *p* = 0.017, Fig. 2*C*). Together, these results demonstrate that cerebellar excitability is significantly reduced after a fatiguing task and relates to milder fatigue perception, but not fatigability.

#### Corticomotor and intracortical excitability after a fatiguing task does not relate to fatigue or fatigability

After a fatigue task, there is a decrease in corticomotor excitability (Brasil-Neto et al., 1993), a phenomenon called postexercise depression (PED) of MEP amplitude (Brasil-Neto et al., 1993; Gandevia, 2001). Thus, we examined whether PED was present after our experimental sessions by measuring MEP amplitude before and after the experimental tasks. We found a significant main effect of TIME (*F*_(1.28,21.90)_ = 17.17, *p* < 0.001) and a TASK × TIME interaction (*F*_(1.47,25)_ = 5.25, *p* = 0.02). *Post hoc* analysis demonstrated that MEP amplitude was significantly reduced at P1 and P2 after the fatigue task. Instead, while MEP amplitude was significantly reduced at P1 after the control task, although to a lesser extent than after the fatigue task, it had returned to baseline levels at P2 (Table 1). These results suggest that, while PED is stronger after fatiguing tasks, it is also present after nonfatiguing ones.

We then investigated whether PED related to fatigue and/or fatigability. To do so, we examined the correlation between MEP_P1_ amplitude and fatigue ratings, MVC after the fatigue task, as well as average TTF. We found that MEP_P1_ amplitude did not correlate with fatigue ratings (*r* = 0.05, *p* = 0.85), MVC (*r* < −0.01, *p* = 0.97), nor TTF (*r* = 0.07, *p* = 0.79). Together, these results suggest that PED does not relate to fatigue nor fatigability and raise the possibility that changes in corticomotor excitability reflect use-dependent excitability changes that relate to exercise intensity rather than to fatigue/fatigability.

Although our observed changes in cerebellar excitability are not likely the result from changes in corticomotor excitability, because we adjusted the MEP amplitude before our measures of CBI, we examined whether PED in M1 and reduced CBI after the fatigue task are related phenomena. We found that MEP_P1_ amplitude did not correlate with reduced CBI (*r* = −0.34, *p* = 0.26), suggesting that reduce CBI and corticomotor excitability occur simultaneously, but are independent processes.

Last, to understand whether reduced cerebellar and corticomotor excitability is also accompanied with global excitability changes, we obtained two measures of intracortical excitability (Table 1): SICI and ICF. Regarding SICI, we found a significant main effect of TIME (*F*_(2,26)_ = 5.19, *p* = 0.013) demonstrating that SICI was significantly reduced immediately after (P1) the experimental tasks but returned to baseline levels (B) after 30 min of recovery (P2). Notably, we did not find a significant TASK × TIME interaction (*F*_(2,26)_ = 1.13, *p* = 0.34), suggesting that SICI changes were comparable between experimental tasks and that fatigue/fatigability did not have a specific effect on intracortical inhibition. On the other hand, regarding ICF, we did not find any significant main effect (*p* > 0.05) nor interaction (*F*_(2,26)_ = 1.02, *p* = 0.38), suggesting that after a fatiguing task ICF remains unchanged.

### Experiment 2: Behavioral consequences of reduced cerebellar excitability after a fatiguing task

**Figure 3.**
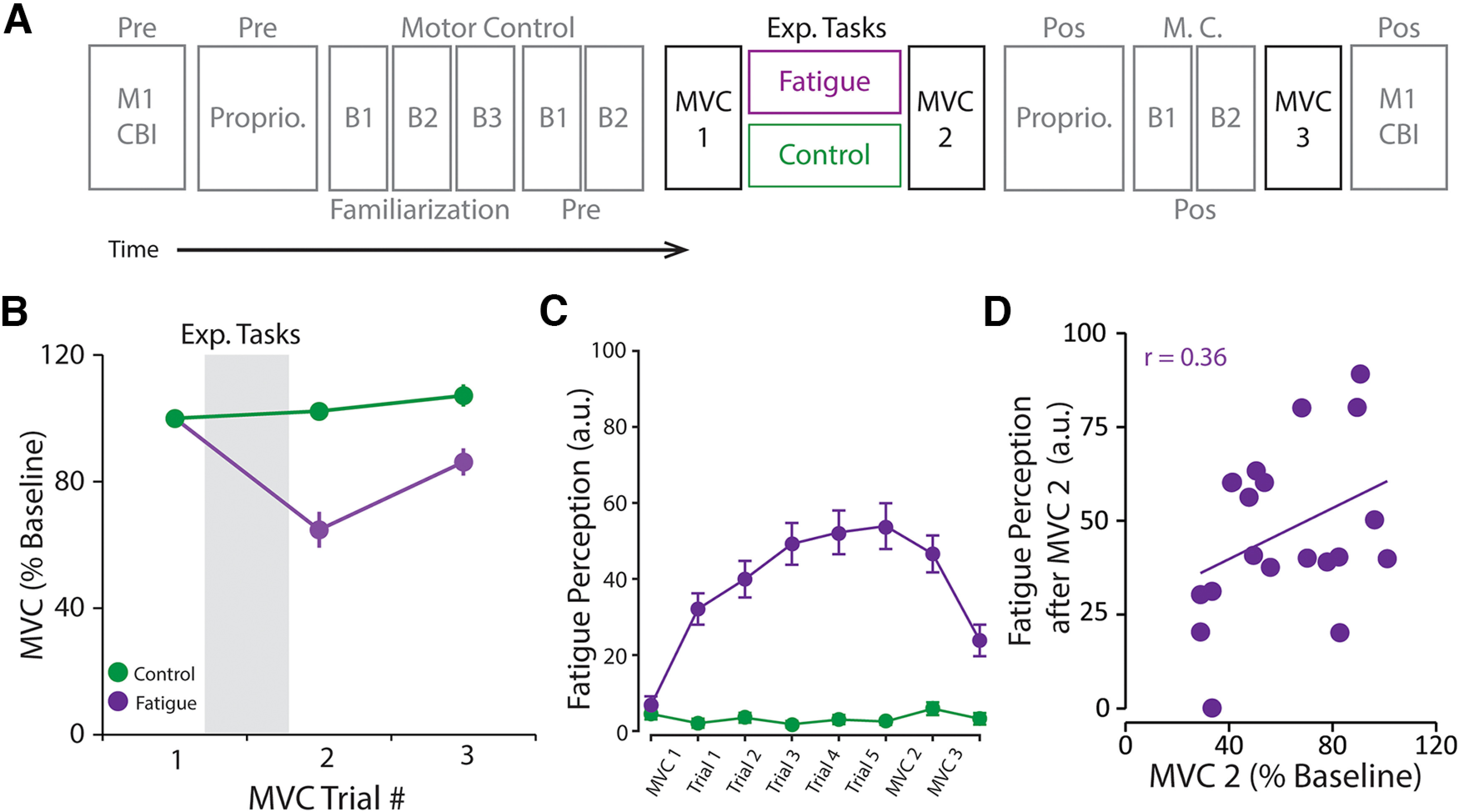
Design of Experiment 2. ***A***, Experiment 2 also consisted of two nonconsecutive sessions separated by at least 24 h. Each session was either a fatigue or a control one, and the order was pseudo-randomized across participants. At the beginning of all sessions, we obtained baseline measures (Pre) of corticomotor excitability (M1), cerebellar brain inhibition (CBI), strength (MVC), as well as performance during a position matching task (proprioception) and a fast reverse-at-target goal-directed task (motor control). Participants then performed one of the experimental tasks (i.e., fatigue or control) where we obtain measures of both fatigue and fatigability. The fatigue task consisted of 5 TTF trials, whereas the control task consisted of 5 constant isometric low-force trials. Right after the experimental tasks, participants repeated the proprioceptive and motor task to examine the effects of fatigue and fatigability on performance of tasks that rely heavily on the cerebellum. Last, we reassessed M1 excitability and CBI at the end of the sessions. ***B***, The fatigue task (purple) significantly reduced participant's MVC throughout the length of the experimental session, whereas MVC remained unchanged after the control task. ***C***, The fatigue task (purple) significantly increased participant's fatigue perception throughout the length of the experimental session, whereas fatigue perception remained low and unchanged after the control task (green). ***D***, Fatigue (perception) and fatigability (i.e., % MVC after the fatigue task) are independent. In time-course result plots, circles represents the average for each session at each time point, and error bars represents the SEM across participants.

Together, Experiment 1 demonstrated that reduced cerebellar excitability after a fatiguing task relates to milder fatigue perception. In Experiment 2, we examined the interaction between reduced cerebellar excitability, fatigue, and performance during two tasks known to engage the cerebellum: (1) a fast reverse-at-target goal-directed task (Bastian, 2006; Casamento-Moran et al., 2015; Casamento-Moran et al., 2020) [to assess the consequences on motor control]; and (2) a position matching task (Bhanpuri et al., 2012, 2013) [to assess the consequences on active proprioception] (Fig. 3*A*).

#### Sample size justification

Using Experiment 1, cerebellar excitability changes after the fatigability task 2, with a medium to large effect size (0.56), a significance of < 0.05, with a power goal of at least 0.80, we required 10 participants to power Experiment 2. However, because of the low tolerability of CBI we recruited a cohort of 20 participants, of which 12 tolerated CBI.

#### Fatigue and fatigability (replication)

As in Experiment 1, we used participants' MVC throughout the experimental sessions to quantify the level of fatigability induced by the experimental tasks (Fig. 3*A*). We found a significant main effect of TASK (*F*_(1,19)_ = 35.59, *p* < 0.001), suggesting lower MVC during the fatigue session compared with the control one. Importantly, we also found a significant TASK × TIME interaction (*F*_(2,38)_ = 18.72, *p* < 0.001; Fig. 3*B*). Again, *post hoc* analysis demonstrated that while baseline strength was similar between sessions, the fatigue task significantly reduced participant's force capacity, whereas the control task did not. When using *Raw MVC* values, we found a significant main effect of TASK (*F*_(1,19)_ = 34.98, *p* < 0.001) and a significant TASK × TIME interaction (*F*_(2,38)_ = 17.31, *p* < 0.001) demonstrating similar patterns.

On the other hand, at baseline, perception of fatigue was similar between the fatigue (7.18 + 2.44) and control (4.93 + 1.5) sessions (*t*_(1,19)_ = −1.06, *p* = 0.30, Fig. 3*C*). In addition, fatigue perception was significantly lower than mild in both experimental sessions (fatigue: *t*_(1,19)_ = −5.91, *p* < 0.001; control: *t*_(1,19)_ = −10.03, *p* < 0.001). However, we found that participants fatigue ratings were significantly higher after the fatigue task compared with the control one (fatigue: 47.04 + 4.98, control: 6.30 + 1.71, *t*_(1,19)_ = 5.73, *p* < 0.001, Fig. 3*C*), once again confirming that fatigue task induced both fatigability and fatigue.

As in Experiment 1, we then assessed whether fatigue related to fatigability after the fatigue task by examining the correlation between fatigue ratings, reduced MVC, and average TTF. We found that neither MVC (*r* = 0.36, *p* = 0.11) nor TTF (*r* = 0.06, *p* = 0.78) was significantly correlated with fatigue, replicating our previous observations that the fatigue and fatigability are independent phenomena.

#### Reduced cerebellar excitability relates to milder fatigue (replication)

**Figure 4.**
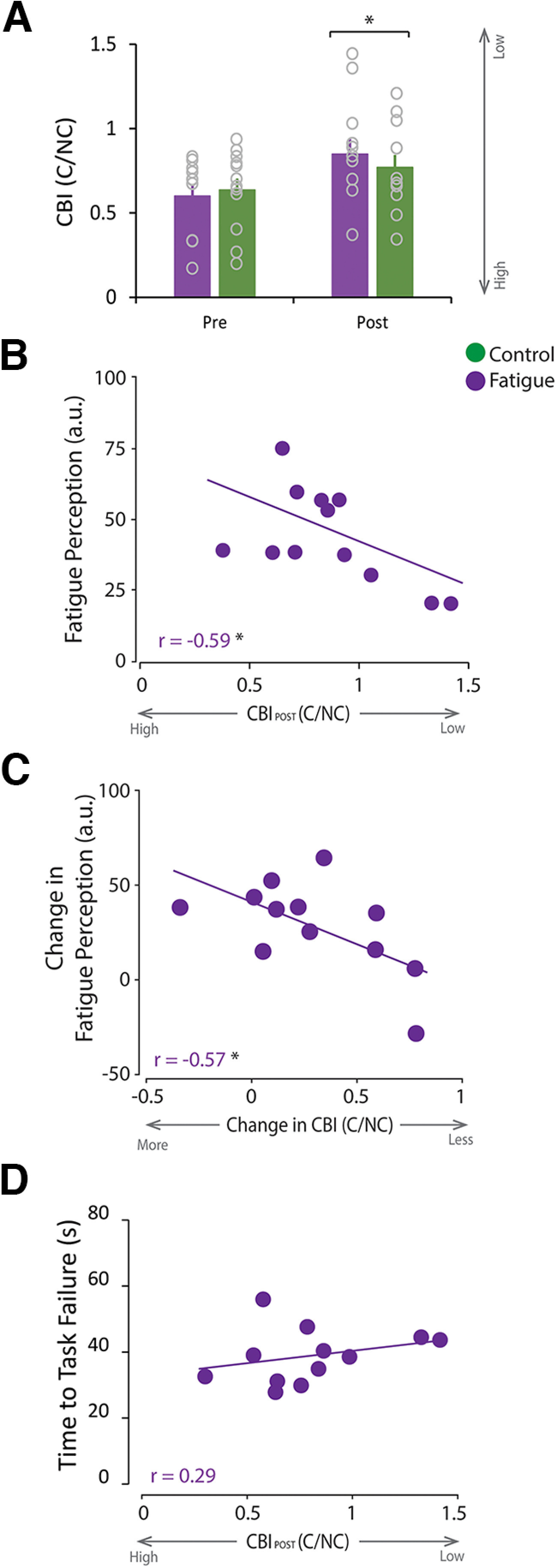
Reduced CBI relates to a milder perception of fatigue in Experiment 2 (Replication). ***A–D***, Purple represents data during the fatigue session. Green represents data during the control session. For all bar graphs, the bar represents the average for each condition at each time point, with individual data represented by the open circles, and the error bar represents the SEM across participants. ***A***, CBI was significantly reduced after both the fatigue and control tasks. ***B***, CBI_Post_ was significantly and negatively correlated with fatigue ratings. ***C***, Change in CBI (Post relative to Pre) was significantly and negatively correlated with the change in fatigue ratings. ***D***, CBI_Post_ did not correlate with TTF (i.e., fatigability). *p < 0.05.

To replicate our observations that reduced cerebellar excitability relates to milder fatigue and is independent from fatigability, we measured CBI at the beginning and end of each experimental sessions (Fig. 3*A*). We found a significant main effect of TIME (*F*_(1,10)_ = 11.16, *p* = 0.007; Fig. 4*A*), suggesting that CBI was lower after both experimental tasks. However, we did not find a significant TASK × TIME interaction (*F*_(1,10)_ = 0.74, *p* = 0.41). Of note, the fatigue-related change in CBI (ΔCBI_Fat_: 0.25) is greater than the known measurement noise of the technique (SDC = 0.081) (Mooney et al., 2021). It is important to highlight that the different experimental designs of Experiments 1 and 2 can contribute to the nonsignificant interaction. In Experiment 1, we assessed CBI immediately before and after the fatigue/control tasks (Fig. 1*A*). However, in Experiment 2, we prioritized the behavioral measures and assessed CBI before and after not only the fatigue/control tasks but also after the performance of cerebellar-dependent tasks (Fig. 3*A*). The performance of cerebellar-dependent tasks can reduce CBI (Jayaram et al., 2011; Schlerf et al., 2012; Spampinato and Celnik, 2017, 2018; Uehara et al., 2018). Thus, the nonsignificant interaction likely reflects the reduction of cerebellar excitability induced by the performance cerebellar-dependent tasks in the control task rather than the lack of effect of fatigue. Indeed, informal *post hoc* analysis revealed that CBI was significantly lower after the fatigue task (*F*_(1,10)_ = 8.19, *p* = 0.017, Fig. 4*A*) and trended toward, but was not significantly lower after the control task (*F*_(1,10)_ = 2.88, *p* = 0.12, Fig. 4*A*). These results replicate the observation of reduced cerebellar excitability a fatigue task and showcase the importance of carefully planning the experimental approach, as it can affect the ability to measure neurophysiological changes that may result from the intervention of interest.

We then examined whether reduced cerebellar excitability relates to fatigue and fatigability by examining the correlation between CBI_Post,_ fatigue ratings, reduced MVC, and average TTF after the fatigue task. We found that CBI_Post_ was significantly and negatively correlated with fatigue ratings (*r* = −0.59, *p* = 0.04; Fig. 4*B*), but not with reduced MVC (*r* = −0.22, *p* = 0.49) nor TTF (*r* = 0.29, *p* = 0.36, Fig. 4*D*). Further, we found that a greater change in CBI was significantly and negatively correlated with a smaller change in fatigue (perception) ratings (*r* = −0.57, *p* = 0.05, Fig. 4*C*). These results replicate, in a separate cohort, our previous observation that reduced cerebellar excitability after a fatiguing task relates to milder fatigue perception and is independent from fatigability.

#### Corticomotor excitability, fatigue, and fatigability

For completeness, we also examined changes in corticomotor excitability (i.e., PED). We did not find any significant main effect of TIME (*F*_(1,10)_ = 3.97, *p* = 0.078) nor TASK × TIME interaction (*F*_(1,10)_ = 0.247, *p* = 0.63, Table 1). It is possible that the absence of PED is a consequence of the time point at which we took the neurophysiological measures. Indeed, these results are in line with the observation that by P2 in Experiment 1 (i.e., 30 min after the experimental task), corticomotor excitability had returned to baseline levels after the control task and was closer to baseline levels after the fatigue task.

To ensure that corticomotor excitability does not relate to fatigue and fatigability, we examined the correlation between MEP_Post_ amplitude and fatigue ratings, average TTF, as well as MVC after the fatigue task. We found that MEP_Post_ amplitude did not correlated with fatigue ratings (*r* = −0.35, *p* = 0.26), TTF (*r* = 0.16, *p* = 0.62) nor MVC (*r* = 0.49, *p* = 0.10). These results demonstrate that corticomotor excitability does not relate to fatigue nor to fatigability, reinforcing the idea that changes in corticomotor excitability reflect use-dependent excitability changes that are independent of fatigue.

#### Cerebellar excitability and motor control

**Figure 5.**
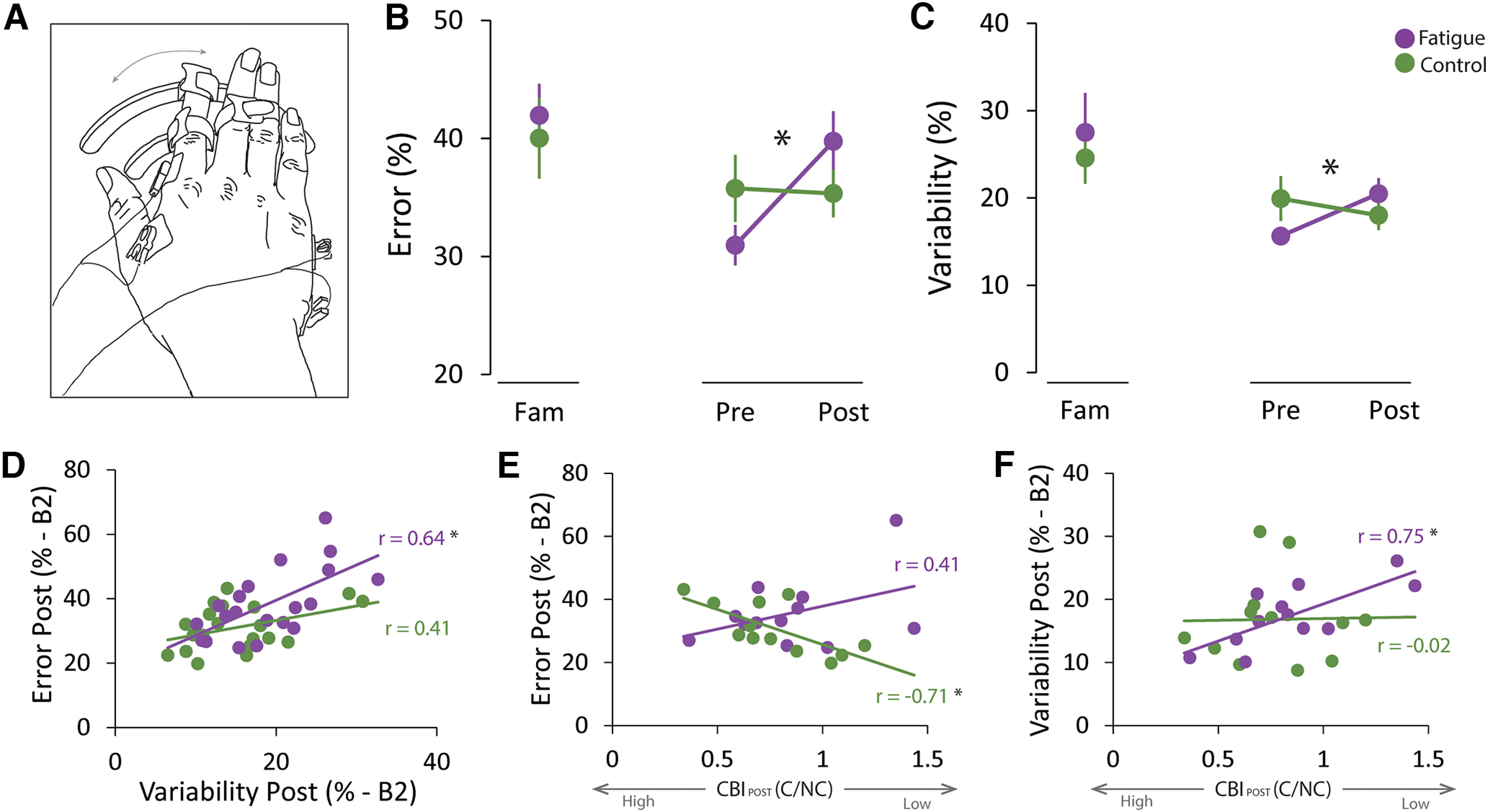
Reduced CBI relates to impaired performance after fatigue. To examine the motor control consequences of reduced cerebellar excitability after a fatiguing task, we used a fast reverse-at-target goal-directed task that requires the preplanned coordination of the antagonistic muscles, which relies on cerebellar function, and minimizes the influence of fatigue-induced tremor on endpoint error. We quantified endpoint error and endpoint variability to examine fatigue-related changes in performance. ***A***, Graphical representation of the positioning of the hands during both the motor control and the proprioception tasks. ***B***, Endpoint error worsen after the fatigue but not the control task. ***C***, Endpoint variability worsens after the fatigue but not the control task. ***D***, Endpoint error during the last motor control block (B2) was significantly correlated with Endpoint variability after the fatigue, but not the control, task. ***E***, Lower endpoint error (i.e., more accuracy) during B2 after the control task correlated with lower CBI. We did not observe this correlation during the fatigue session. ***F***, Greater endpoint variability during B2 after the fatigue task correlated with lower CBI. We did not observe this correlation during the control session. *p < 0.05. In time-course result plots, circles represents the average for each session at each time point, and error bars represents the SEM across participants.

To examine whether the reduced excitability related to the adverse effects of fatigue on motor control, we quantified endpoint error and variability during a fast reverse-at-target goal-directed task that requires the preplanned coordination of the antagonistic muscles (Casamento-Moran et al., 2017) and relies on cerebellar function (Lang and Bastian, 1999; Köster et al., 2002; Casamento-Moran et al., 2020). For both endpoint error and endpoint variability, we found a TASK × TIME interaction (endpoint error: *F*_(1,19)_ = 9.84, *p* = 0.005; Fig. 5*B*; endpoint variability: *F*_(1,19)_ = 5.51, *p* = 0.03; Fig. 5*C*). *Post hoc* analysis demonstrated that both metrics worsen after the fatigue but not after the control task, suggesting that motor control is impaired after a fatiguing task. Interestingly, we found that error and variability were significantly correlated after the fatigue (*r* = 0.64, *p* = 0.003) but not the control (*r* = 0.41, *p* = 0.07; Fig. 5*D*) task.

To understand whether the association between reduced cerebellar excitability and impaired motor control, we examined whether endpoint error or variability related to CBI after the fatigue task. While endpoint error did not correlate with CBI (*r* = 0.41, *p* = 0.19, Fig. 5*E*), we found that greater endpoint variability was significantly correlated with lesser CBI (*r* = 0.75, *p* = 0.005, Fig. 5*F*), a finding consistent with prior studies (Schlerf et al., 2015). Furthermore, we assessed whether the correlation between CBI, endpoint error, and endpoint variability was significantly different between the fatigue and control sessions. We found that, while the correlation between endpoint error and variability was significant in the fatigue, but not the control session (Fig. 5*C*), these correlations were not statistically different from each other (*z* = 0.94; probability = 0.34). In contrast, the correlation between CBI and behavior was significantly different between the fatigue and control sessions (error: *z* = 3.86, probability < 0.001, Fig. 5*D*; variability: *z* = 2.89, probability = 0.004, Fig. 5*E*).

#### Fatigability and motor control

To examine whether participants may have prioritized the motor task over the fatiguing task, we performed bivariate correlations between our fatigue (ratings), fatigability (MVC_Post,_ TTF), and motor control (endpoint error and variability) measures. The correlations were nonsignificant for both endpoint error (fatigue: *r* = −0.21, *p* = 0.38; MVC_Post_: *r* = 0.01, *p* = 0.95; TTF: *r* = 0.39, *p* = 0.09) and endpoint variability (fatigue: *r* = −0.14, *p* = 0.57; MVC_Post_: *r* = 0.04, *p* = 0.86; TTF: *r* = 0.25, *p* = 0.99). These results demonstrate that there is no direct relationship between performance (and perception) during the fatigue and “motor control” tasks, suggesting that participants did not prioritize one over the other.

#### Proprioception

**Figure 6.**
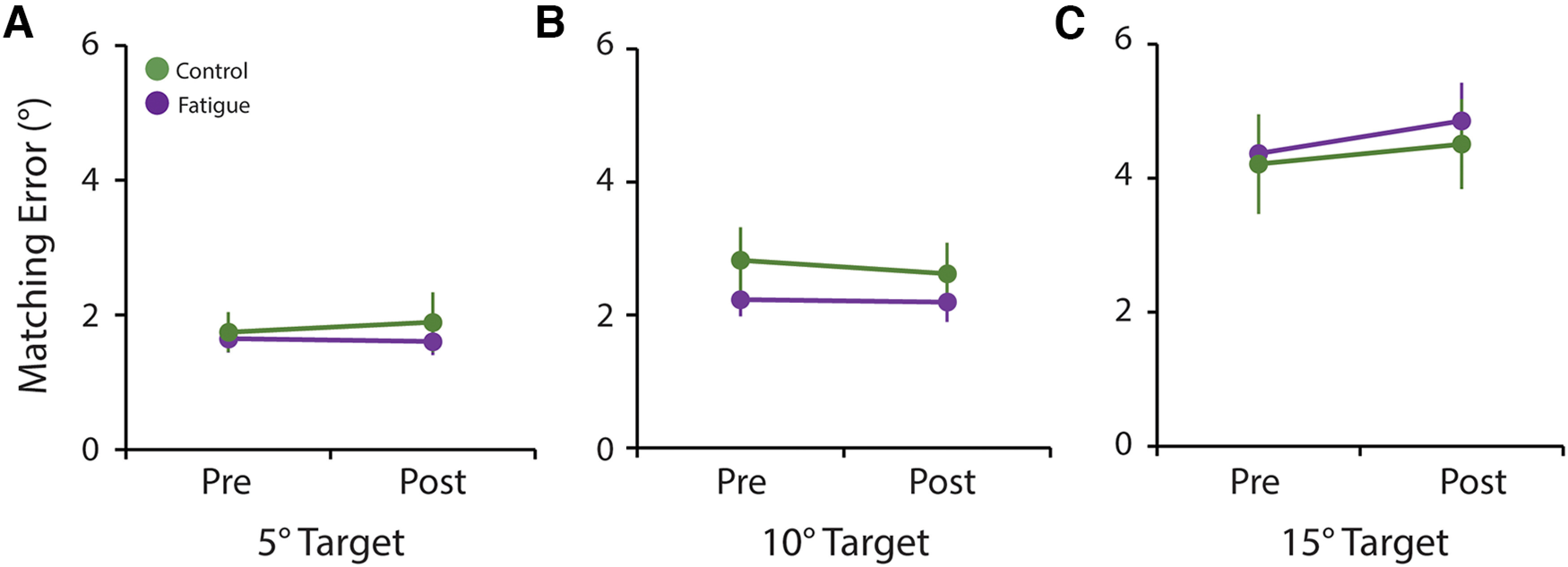
Lack of proprioceptive differences after fatigue. ***A–C***, Purple represents data during the fatigue session. Green represents data during the control session. Position matching error was similar before and after the fatigue and control tasks at all target sizes (***A***, 5°; ***B***, 10°; ***C***, 15°). Circles represents the average for each session at each time point and error bars represents the SEM across participants.

To examine the proprioceptive consequences of reduced cerebellar excitability after a fatiguing task, we quantified matching error during an active position matching (Bhanpuri et al., 2012, 2013). We did not find any significant main effect (all *p* > 0.5) nor TASK × TIME interaction (all *p* > 0.4), indicating that active proprioception is not impaired after a fatiguing task (Fig. 6).

## Discussion

We investigated the role of the cerebellum in fatigue by examining whether cerebellar excitability is modulated after a fatiguing task and whether it relates to fatigue perception. We found that decreased cerebellar excitability correlated with milder fatigue perception such that the larger the reduction in CBI, the smaller the increase in fatigue perception. While correlation does not imply causation, we interpret the current results to suggest that changes in cerebellar excitability after a fatiguing task contribute to modulate fatigue perception. Our results align with the idea that, under fatiguing conditions, deactivation of cortical and subcortical areas calibrates the sensorimotor state to help maintain homeostasis (Hogan et al., 2020) and raise the possibility that the cerebellum plays a significant role in such calibration.

Fatigue is thought to arise from the inability to maintain bodily homeostasis (Stephan et al., 2016; Kuppuswamy, 2017; van der Schaaf et al., 2018). In parallel, the “universal cerebellar transform” (Schmahmann et al., 2019) theory states that the cerebellum makes a consistent computation across all domains (sensorimotor, cognitive, limbic). By integrating internal representations with external stimuli, the cerebellum helps maintain the different behaviors around a homeostatic baseline to optimize performance according to context (Barash et al., 1999; Schmahmann et al., 2019). It is possible, therefore, that under fatiguing conditions, the cerebellum helps maintain homeostasis by updating the priors regarding the sensorimotor and interoceptive state (Schutter, 2016), which in turn minimizes fatigue.

The cerebellum plays a key role in optimizing motor control, kinesthesia, and learning (Bastian, 2006; Manto et al., 2012). Thus, we also investigated whether the cerebellum is capable of simultaneously minimizing fatigue and optimizing performance or whether these two processes compete for cerebellar resources. We measured cerebellar excitability, fatigue, and performance during a ballistic goal-directed task. We chose this ballistic task because it emphasizes endpoint accuracy, minimizes the effect of fatigability-related tremor on performance, and it is used to characterize dysmetria (the cardinal sign of cerebellar dysfunction) (Manto, 2009) in clinical populations (Casamento-Moran et al., 2015; Casamento-Moran et al., 2020). Since motor control (Gates and Dingwell, 2008; Missenard et al., 2008; Lohse and Sherwood, 2012; Monjo et al., 2015; Hunter, 2018; Bächinger et al., 2019) and learning (Branscheidt et al., 2019) are impaired after a fatiguing task, we hypothesized that reduced cerebellar excitability will relate to milder fatigue, but compromised performance. As hypothesized, we replicated the observation that lower cerebellar excitability correlated with milder fatigue and found that lower cerebellar excitability correlated with greater movement variability after the fatiguing task. In parallel, we found that endpoint error and variability were significantly related after the fatigue but not the control task. The myriad of neuromuscular and metabolic changes (Bigland-Ritchie and Woods, 1984; Gandevia, 2001; Hunter et al., 2004, 2008; Potvin and Fuglevand, 2017; Hunter, 2018) that the nervous system undergoes during/after fatiguing tasks, leads to the selection of different neuromuscular strategies to execute actions, such as increasing the neuromuscular drive or altering cocontraction (Bigland-Ritchie and Woods, 1984; Missenard et al., 2008; Santos et al., 2010; Turpin et al., 2011). The usage of these compensatory strategies could lead to a more unpredictable and variable performance and inaccuracy. Alternatively, it is possible that under fatiguing conditions the nervous system sacrifices consistency/accuracy as a mean of energy conservation. A previous study that focused on understanding the role of fatigue in motor control found that, while participants could have increased muscular cocontraction to improve performance, they did not do so (Missenard et al., 2008). The authors proposed that the nervous system could change the respective importance assigned to motor control and energy expenditure during fatiguing conditions to save energy rather than optimize performance (Missenard et al., 2008). Our results support and expand this idea by demonstrating that the cerebellum may help regulate both performance and fatigue. We propose that the cerebellum optimizes performance under rested circumstances; however, during/after a significant physical challenge and lower energetic resources, performance in a task that has no repercussions for the homeostatic state is sacrificed, and resources are geared toward fatigue-related processes. It is worth highlighting that TMS limits our ability to examine whether other brain regions also contribute to poor motor control after a fatiguing task. Future studies could use whole-brain neuroimaging approaches to examine whether changes throughout the cerebello-thalamo-cortical loop or other brain networks are involved.

A seemingly inconsistent result is the observation that cerebellar excitability is uncorrelated with decreased force capacity while simultaneously correlated with increased motor variability. However, these are two different performance measures, in which the cerebellum may play a different role. Evidence from cerebellar disorders, such as spinocerebellar ataxia (SCA) Type 6, a cerebellar disease characterized by “pure” cerebellar degeneration (Sasaki et al., 1998; Solodkin and Gomez, 2012), suggest that, while the cerebellar degeneration impairs motor control (Casamento-Moran et al., 2015; Yacoubi et al., 2020), it does not affect maximal force capacity (Casamento-Moran et al., 2015). This may be the case because, while fine motor control requires the precise coordination of the involved muscles, function attributed to the cerebellum (Manto, 2009; Diedrichsen and Bastian, 2013), maximal force generation is coarser and may rely on other spinal and/or subcortical systems (Škarabot et al., 2022; Shalit et al., 2012; Lodha et al., 2018; Glover and Baker, 2022).

Fatigue is a highly prevalent but understudied symptom in cerebellar disorders, such as SCA (Brusse et al., 2011), autism (Raymaker et al., 2020), and schizophrenia (Waters et al., 2013). It is possible that, when injured or dysfunctional, the cerebellum losses its ability to adapt to the changing interoceptive conditions, contributing to bodily dyshomeostasis, and fatigue. Future studies should test this hypothesis and examine the role of the cerebellum in pathologic fatigue.

Our observations trigger an important question: what processes is the cerebellum modulating to minimize fatigue? Growing evidence suggests that interindividual differences in interoceptive abilities (e.g., accuracy, sensibility, and awareness) (Garfinkel et al., 2015) relate to the strength of emotional experiences, such as anxiety (Critchley et al., 2004). If fatigue reflects an affective response to dyshomeostasis (Stephan et al., 2016), then a plausible hypothesis is that the cerebellum could modulates individual's interoceptive abilities as the means to regulate fatigue. In other words, by modulating the accuracy, sensibility, and awareness of fatigue-related interoceptive signals, the cerebellum could regulate the intensity of perceived fatigue. Future studies should examine whether interoceptive abilities contribute to the strength of fatigue perception, and if so, what is the specific modulatory role that the cerebellum plays in fatigue-related interoception.

Mild inflammatory processes increase fatigue perception (Stefanov et al., 2020). Repeated effortful exertions, like the ones used in this study, elicit a cascade of metabolic and inflammatory process in the muscle (Hunter, 2018). Theoretically, the cerebellum could modulate how individuals sense the metabolic/inflammatory state of the muscle directly via the cerebello-thalamo-cortical loop or indirectly via second-order synapses (thalamic or striatal) reaching the insular cortex (Ghaziri et al., 2018). It is important to mention that, while our study demonstrates a modulatory role of the cerebellum in fatigue perception, the use of TMS prevents us from examining the role of other subcortical areas in fatigue. As mentioned above, the thalamus and insular cortex are two regions that could contribute to the modulation of fatigue perception. The thalamus is thought to be involved in the pathogenesis of pathologic fatigue, since individuals with multiple sclerosis (Capone et al., 2020) or traumatic brain injury (Clark et al., 2018), who suffer fatigue, exhibit thalamic structural and functional changes. On the other hand, the insular cortex, known for its key role in the processing and sensing of interoceptive information (Craig, 2009), could contribute to the perception of fatigue since, for example, increased effective connectivity between specific nodes of the insula mediates the association between inflammation and fatigue (Stefanov et al., 2020). Indeed, the insula was proposed to be part of a “cognitive fatigue network” (Chen et al., 2020; Wylie et al., 2020). Future studies should examine the role of the thalamus and insular cortex in the perception of physical fatigue and investigate how these areas interact with the cerebellum to modulate fatigue.

### Fatigue, fatigability, motor control, and kinesthesia are independent phenomena

Fatigue should not be confused with fatigability. While fatigue refers to the subjective sensation of weariness, fatigability refers to objective decrements in performance (either physical or cognitive) over time (Kluger et al., 2013). Here we provide supportive evidence to the idea that fatigue and fatigability are independent phenomena that relate to different neurophysiological mechanisms. We propose that, while fatigue perception could reflect an affective response to dyshomeostasis, fatigability could reflect the residual capacity of the sensorimotor system that results from the combination of level of dyshomeostasis and the compensatory processes triggered to offset such dyshomeostasis and maintain performance (i.e., the increased neuromuscular drive [compensatory process] that counteracts for the reduced muscle force capacity [dyshomeostasis]) (Hunter et al., 2004, 2008; Potvin and Fuglevand, 2017; Hunter, 2018).

However, and as done in this study, fatigability can be a useful experimental manipulation to understand fatigue in health and disease (Kluger et al., 2013; Penner and Paul, 2017). If fatigability is used to understand fatigue, it is essential that experimental designs measure fatigue alongside with fatigability. Unfortunately, most studies only investigate the factors that contribute to fatigability because they do not directly quantify fatigue. For example, previous neuroimaging studies used a similar fatiguing task as the one used here but did not survey fatigue (Sosnoff and Newell, 2006; Nakagawa et al., 2013; Hou et al., 2016). These studies demonstrated deactivation of different brain areas, including the cerebellum, but assumed that the observed deactivation related to greater fatigue/fatigability without disentangling the two (Sosnoff and Newell, 2006; Nakagawa et al., 2013; Hou et al., 2016). Thus, our results suggest that we must measure fatigue and fatigability to understand and dissociate the significance of the neural changes that parallel these phenomena. We also found that, despite motor control being detrimentally affected by our fatigue task, it did not correlate with fatigue nor fatigability. These results suggest that, while motor control worsens after fatiguing task, the mechanisms of such effect are different from that of increased fatigue and fatigability.

Last, we also observed that kinesthesia was not affected by the fatigue task nor decreased cerebellar excitability. These results suggest that optimal sensorimotor calibration and/or homeostatic regulation under fatiguing conditions may require a reliable estimation of our body in space. While we can afford to sacrifice motor control in a task that has no repercussions for our homeostatic state, we cannot afford to lose our understanding of where our body is in space. Doing so could potentially push us toward further dyshomeostasis and injury.

### Limitations and future directions

CBI reflects the connectivity between the cerebellum and the primary motor cortex (M1). Specifically, it is the result of Purkinje cell activation by the CS, leading to inhibition of the deep cerebellar nuclei, which in turn have an excitatory connection with M1 via thalamic nuclei (Ugawa et al., 1995, 1997). Thus, although we are interpreting the reduction in CBI to reflect a reduction in cerebellar excitability, we cannot rule out nor measure the thalamic excitability changes that contribute to reduced CBI and milder fatigue. Furthermore, the use of TMS limits our ability to examine whether other brain regions modulate fatigue. Future studies should use whole-brain neuroimaging approaches to examine whether changes throughout the cerebello-thalamo-cortical loop or other brain networks modulate fatigue.

Another intrinsic limitation of using TMS as a technique to assess cerebellar excitability is that participants can find the stimulation over the cerebellum quite uncomfortable (Spampinato et al., 2020; Rurak et al., 2022). This prevents us from assessing CBI changes in bigger sample sizes. Despite its low tolerability, CBI as a technique has low measurement error (∼15%) within and between sessions and its SDC does not change significantly with sample sizes >10 participants (Mooney et al., 2021).

Last, we observe that some individuals show CBI values >1 after the fatigue task, which may reflect facilitation of the MEP with cerebellar conditioning. We are unaware of a specific physiological mechanism underlying this observation and propose that these values may reflect a reduction in activity of Purkinje cells and noise in the MEP measurement.

In conclusion, we aimed to understand how cerebellar excitability is modulated after a fatiguing task and its association with fatigue and performance. We demonstrated that reduced cerebellar excitability after a fatiguing task relates to milder fatigue but worse motor control. These results suggest that decreased cerebellar excitability minimizes fatigue at the expense of performance. We propose that the cerebellum may help regulate both performance and fatigue, but modulation of fatigue interplays with optimal motor control. Future work should test this hypothesis to better understand fatigue and develop effective treatment and rehabilitation strategies for this common and debilitating symptom.
